# Neuroprotective Effect of Caffeic Acid Phenethyl Ester in A Mouse Model of Alzheimer’s Disease Involves Nrf2/HO-1 Pathway

**DOI:** 10.14336/AD.2017.0903

**Published:** 2018-08-01

**Authors:** Fabiana Morroni, Giulia Sita, Agnese Graziosi, Eleonora Turrini, Carmela Fimognari, Andrea Tarozzi, Patrizia Hrelia

**Affiliations:** ^1^Department of Pharmacy and Biotechnology, Alma Mater Studiorum, University of Bologna, Bologna, Italy; ^2^Department for Life Quality Studies, Alma Mater Studiorum, University of Bologna, 47900 Rimini, Italy

**Keywords:** caffeic acid phenethyl ester, neuroprotection, Aβ oligomers, Alzheimer’s disease, Nrf2, oxidative stress

## Abstract

Alzheimer’s disease (AD) is a progressive pathology, where dementia symptoms gradually worsen over a number of years. The hallmarks of AD, such as amyloid β-peptide (Aβ) in senile plaque and neurofibrillary tangles, are strongly intertwined with oxidative stress, which is considered one of the common effectors of the cascade of degenerative events. The endogenous nuclear factor erythroid 2-related factor 2 (Nrf2) is the "master regulator" of the antioxidant response and it is known as an indicator and regulator of oxidative stress. The present study aimed to determine the potential neuroprotective activity of caffeic acid phenethyl ester (CAPE), a polyphenolic compound abundant in honeybee, against the neurotoxicity of Aβ_1-42_ oligomers (AβO) in mice. An intracerebroventricular (i.c.v.) injection of AβO into the mouse brain triggered increased reactive oxygen species levels, neurodegeneration, neuroinflammation, and memory impairment. In contrast, the intraperitoneal administration of CAPE (10 mg/kg) after i.c.v. AβO-injection counteracted oxidative stress accompanied by an induction of Nrf2 and heme oxygenase-1 via the modulation of glycogen synthase kinase 3β in the hippocampus of mice. Additionally, CAPE treatment decreased AβO-induced neuronal apoptosis and neuroinflammation, and improved learning and memory, protecting mice against the decline in spatial cognition. Our findings demonstrate that CAPE could potentially be considered as a promising neuroprotective agent against progressive neurodegenerative diseases such as AD.

Alzheimer’s disease (AD) is currently the most prevalent form of senile dementia, which affects millions of people worldwide. The major pathological hallmarks of AD include neurofibrillary tangles (NFTs), senile (neuritic) plaques (SPs) and synapse loss. Amyloid β-peptide (Aβ) is the principal constituent of the SP and is proposed, mainly on a genetic basis, as being central to the pathogenesis of this disorder [1]. Since 1992, the amyloid cascade hypothesis has played a crucial role in explaining the etiology and pathogenesis of AD [2]. Several studies have suggested that Aβ can diffuse readily through the brain parenchyma and activate a cascade of pathogenic events such as the culmination of neuronal apoptosis/necrosis [3], induction of oxidative stress [4] and neuroinflammation [5] in the cortex and hippocampus, which are two of the affected brain regions in AD [6]. The neurotoxic role of soluble amyloid-beta oligomers (AβO) that begin to accumulate in the human brain approximately 10 to 15 years before the appearance of clinical symptoms has been highlighted. Many reports indicate that soluble AβO correlate with memory deficits in AD models and humans [7, 8]. Although soluble amyloid species are recognized triggers of the disease, no therapeutic approach is able to stop it.

Several studies suggest that oxidative stress, known as an imbalance between free radicals and endogenous antioxidant defenses, is a crucial and early feature in the pathogenesis of neuronal damage in AD [9, 10]. The brain is particularly vulnerable to oxidative stress, despite accounting for only 2% of the body’s weight, it consumes 20% of the inspired oxygen, and its membranes are especially sensitive to oxidative damage because of their high content of polyunsaturated fatty acids. Reactive oxygen species (ROS) are particularly active in the brain and due to the presence of excitatory amino acids and neurotransmitters, neuronal tissue is a source of ROS. It has been observed that the entorhinal cortex and CA1 region of the hippocampus are the two most susceptible cerebral regions to oxidative stress [11]. Mitochondrial damage in AD could lead to excessive generation of ROS and lowered ATP production [12]. Research studies demonstrated that interaction between oxidative stress and neuroinflammation leads to Aβ generation [13-15]. These two factors may interact and amplify each other in a vicious cycle of toxicity, leading to neuronal dysfunction, cell dysfunction, and finally cell death.

The nuclear factor erythroid 2-related factor 2, also known as Nrf2, is a member of the cap‘n’collar family of transcription factors and performs a wide range of functions in a variety of organs, including the brain. Nrf2 is the "master regulator" of the antioxidant response, modulating the expression of hundreds of genes, including not only the familiar antioxidant enzymes, but also large numbers of genes that control apparently unrelated processes such as immune and inflammatory responses, tissue remodeling and fibrosis, carcinogenesis and metastasis, and even cognitive dysfunction and addictive behavior [16]. Translocation of Nrf2 into the nucleus, after its release from Keap1 in the cytoplasm, is crucial for the activation of the transcription of numerous protective genes. Insufficient Nfr2 activation in humans has also been linked to chronic diseases such as AD [17]. Thus, stimulating Nrf2 appears to be a promising method for reducing the level of neurodegeneration. Current data indicate that a sophisticated network of signaling mechanisms is involved in Nrf2 regulation.

A growing number of bioactive compounds have been found to be capable of igniting the Nrf2 pathway and preserving the Central Nervous System (CNS) against various insults and damage. Caffeic acid phenethyl ester (CAPE) is a compound abundant in honeybee propolis. Several studies have demonstrated its anti-inflammatory, antiviral, antioxidant and antitumor properties [18-20]. Its beneficial effects against neurodegenerative diseases have also been suggested [21, 22]. CAPE is able to protect blood-brain barrier (BBB) in rodent model of traumatic brain injury [23], prevent neonatal hypoxic-ischemic brain injury [21] and attenuate dopaminergic neuronal loss in 6-OHDA Parkinson’s model [24]. Scapagnini et al. reported that CAPE is a potent inducer of heme oxygenase-1 (HO-1) in astroglial cells and in neurons and it is capable of transcriptionally activating a gene battery that also includes phase II detoxifying enzymes, through the Nrf2 pathway [25]. Moreover, its capacity to cross BBB was demonstrated by Silva et al. [26] using parallel artificial membrane permeability assay (PAMPA), which is a high throughput technique developed to predict passive permeability through biological membranes. However, to the best of our knowledge, there is hardly any report in the literature regarding the effect of CAPE against Aβ-induced toxicity. Interestingly, Kumar et al. [27] demonstrated that CAPE administration ameliorated intracerebroventricolar (i.c.v.) injection of streptozotocin -induced dementia through the attenuation of oxidative stress and inflammation.

Therefore, this study explores the effect of CAPE on AD pathology and cognitive functions against Aβ_1-42_O-induced dementia in mice.

## MATERIALS AND METHODS

### Animals

Male C57Bl/6 (9 weeks old, 25-30 g body weight at the beginning of the experiment; Harlan, Milan, Italy) mice were housed under 12 h light/12 h dark cycle (lights on from 7:00 a.m. to 7:00 p.m.) with free access to food and water in a temperature- and humidity-controlled room. Briefly, all experiments were carried out in accordance with Directive 2010/63/EU and Directive 86/609/CEE and approved by the corresponding committee at the University of Bologna (PROT. n. IX/77 2013). Care was taken to minimize the number of experimental animals and to take measures to limit their suffering. Mice were allowed to acclimatize for at least 1 week before the start of experiments.

### Experimental design

**Figure 1:**
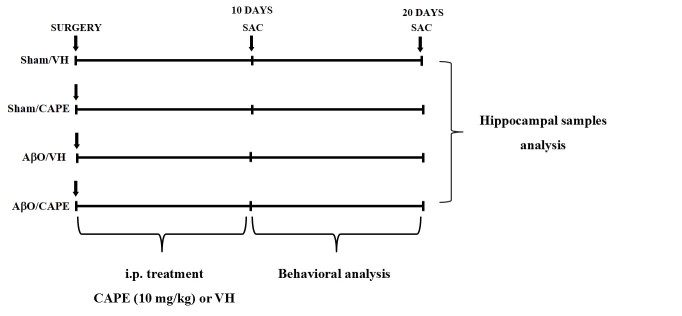
Experimental protocol and CAPE treatment schedule Mice received i.p. injection of CAPE (10 mg/kg) for 10 days. Animals were sacrificed 10 or 20 days after Aβ_1-42_O injection.

The experimental protocol was based on the unilateral stereotaxic i.c.v. injection of A_β1-42_O. Animals were randomly divided into four major groups (n=20/group) as follows: AβO/VH; AβO/CAPE; sham/VH; sham/CAPE. Two groups received an i.c.v. injection of Aβ_1-42_O, while the other two received the same amount of saline solution (sham groups). One hour after brain lesion, we started intraperitoneal (i.p.) administration of 10 mg/kg of CAPE (Lkt Laboratories, St. Paul, MN, USA) or vehicle (VH, saline) in both lesioned and sham mice. The dose injected was selected on the basis of previous studies [28, 23]. We injected mice everyday once a day for 10 days. At the end of the treatment half the group was sacrificed to proceed with biomolecular analysis while the other animals underwent behavioral assessment before the sacrifice. Animals were deeply anesthetized and sacrificed by cervical dislocation to perform immunohistochemistry, neurochemical, and molecular analysis (for experimental design see Fig. 1).

### Aβ oligomers preparation and injection

Aβ_1-42_ peptides (AnaSpec, Fremont, CA, USA) were first dissolved in hexafluoroisopropanol to 1 mg/mL, sonicated, incubated at room temperature for 24 h and lyophilized. The resulting unaggregated Aβ_1-42_ film was dissolved with sterile dimethylsulfoxide to a final concentration of 1 mM and stored at -20 °C until use. The Aβ_1-42_ aggregation to oligomeric form was prepared as described previously by Tarozzi et al. [29]. Briefly, Aβ_1-42_ stock was diluted into phosphate buffer saline (PBS) at 40 μM and incubated at 4 °C for 48 h to enhance oligomer formation [30, 31]. Six µL of Aβ_1-42_O (40 μM) were injected i.c.v., using a stereotaxic mouse frame (myNeuroLab, Leica-Microsystems Co, St. Louis, MO, USA) and a 10 µL Hamilton syringe, at a rate of 0.5 mL/min. The needle was left in place for 3 min after the injection before slow retraction, followed by cleaning and suturing of the wound. Sham mice received the equivalent volume of saline into the ventricle. The injection was performed at the following co-ordinates: AP: +0.22, ML: +1.0, DV: -2.5, with a ?at skull position.

### Behavioral analysis

All tests were carried out between 9.30 a.m. and 3.30 p.m. Animals were transferred to the experimental room at least 1 h before the test in order to let them acclimatize to the test environment. All scores were assigned by the same observer who was unaware of the animal treatment.

### Morris Water Maze (MWM) test

The apparatus used for the MWM task was a circular plastic tank (1.0 m diameter, 50 cm height) filled with water and milk maintained at 22˚C. The maze was located in a room containing several simple visual, extra-maze cues that were constant throughout the study. A transparent platform was set inside the tank and its top was submerged 1.5 cm below the water surface in the center of one the four quadrants of the maze. The movements of the animal in the tank were monitored with a video tracking system (EthoVision, Noldus, The Netherlands). For each training trial, the mouse was put into the pool at one of the four positions, the sequence of the positions being selected randomly. The platform was located in a constant position throughout the test period in the middle of one quadrant. In each training session, the latency to escape onto the hidden platform was recorded. If a mouse failed to find the platform within 60 s, it was manually guided to the platform and allowed to remain there for 10 s. After the trial, each mouse was placed in a holding cage under a warming lamp for 25 s until the start of the next trial. Training was conducted for 5 days, four times a day. On day 6, a single probe trial was performed, which consisted of a 60 s free swim in the pool without the platform. The parameters measured during the probe trial included escape latency, time spent in the opposite quadrant to the platform zone, and total distance from the platform.

### Novel Object Recognition (NOR) test

This task is based on the spontaneous tendency of rodents to explore novel objects [32]. The test was performed in an apparatus made of a white Plexiglass box (60 × 60 × 30 cm) with the ?oor divided into four identical squares in a dim room. Mice were placed in the empty box for 5 min 24 h prior to exposure to objects, in order to habituate them to the apparatus and test room. Twenty-four hours after habituation, mice were acclimated in the test room for 1 h before the beginning of the sessions. Firstly, mice completed an acquisition trial (24 h after habituation) that consisted of leaving the animals in the apparatus containing two identical objects (A and A’). After a 24 h retention interval, the mice were put back into the arena and exposed to the familiar object (A) and to a novel object (B). In all sessions, each mouse was always placed in the apparatus facing the wall and allowed to explore the objects for 5 min, after which the mouse was returned to its home cage. Behavior was recorded by a video camera mounted vertically above the test arena and analyzed using appropriated video-tracking software (EthoVision, Noldus). Between trials, the apparatus was cleaned with 5% ethanol solution to eliminate animal clues. The light inside the apparatus was maintained at a minimum to avoid any anxiety behavior. A recognition index, a ratio of the amount of time spent exploring the novel object over the total time spent exploring both objects, was used to measure cognitive function.

### Tissue preparation for immunohistochemistry and neurochemical analysis

Ten and 20 days after Aβ_1-42_O injection, mice were deeply anesthetized and sacriﬁced by cervical dislocation. The brains were removed and the left hemisphere of each animal was immersed in a 4% ﬁxative solution of paraformaldehyde (Santa Cruz Biotechnology, Dallas, TX, USA) for 48 h. Right hemispheres were rapidly removed, and the hippocampi were dissected in an ice-cold plastic dish. Samples were then snap-frozen in liquid nitrogen, and kept at -80°C until analysis.

Tissues were homogenized in lysis buffer (50 mM Tris, pH 7.5, 0.4% NP-40, 10% glycerol, 150 mM NaCl, 10 µg/mL aprotinin, 20 µg/mL leupeptin, 10 mM EDTA, 1 mM sodium orthovanadate, 100 mM sodium ?uoride), and the cytoplasmic fraction was kept at -20°C until use. The protein in nuclear fraction was extracted using the Nuclear Extract kit (Actif Motif, Carlsbad, CA, USA). Cytoplasmic and nuclear protein concentration was determined by the Bradford method [33].

### Determination of redox status

The redox status, in terms of reactive oxygen species (ROS) formation, was measured as described previously [34], based on the oxidation of 2’7’-dichlorodihydro?uorescein diacetate (DCFH-DA) to 2’7’-dichloro?uorescein (DCF). Brie?y, the reaction mixture (60 μL) containing 2 mg/mL of DCFH-DA was incubated for 30 min to allow the DCFH-DA to be incorporated into any membrane bound vesicles and the diacetate group to be cleaved by esterases. After 30 min of incubation, the conversion of DCFH-DA to the ?uorescent product DCF was measured using a microplate reader (GENios, TECAN®, Mannedorf, Switzerland) with excitation at 485 nm and emission at 535 nm. Background ?uorescence (conversion of DCFH-DA in the absence of homogenate) was corrected by the inclusion of parallel blanks. Values were normalized to protein content and expressed as fold increase of the mean of ?uorescence intensity arbitrary units (UF) of each group compared to the sham group.

### Determination of glutathione (GSH) content

GSH content was assessed using the protocol described earlier [35]. Brie?y, aliquots of 50 μL of samples were precipitated with 100 μL of sulfosalicylic acid (4%). The samples were kept at 4 °C for at least 1 h and then subjected to centrifugation at 3000 rpm for 10 min at 4 °C. A volume of 25 μL of the assay mixture and 50 μL of 5-5’-dithiobis (2-nitrobenzoic acid) (4 mg/mL in phosphate buffer, 0.1 M, pH 7.4) was made up to a total volume of 500 μL. The yellow color that developed was read immediately at 412 nm (GENios, TECAN®) and results were calculated using a standard calibration curve. Values are expressed as fold increase of GSH mmol/mg content in each group compared to the sham group.

### Determination of Nrf2 activation

The Nrf2 activation was assessed using an ELISA kit (TransAM® Kit, Active Motif) according to manufacturer’s instructions. An ELISA-based assay consisting of an immobilized oligonucleotide containing the ARE consensus-binding site (5′-GTCACAGTG ACTCAGCAGAATCTG-3′) was used to measure Nrf2 DNA binding activity. Nrf2 from 20?μg of nuclear extract was allowed to bind to the ARE on 96-well plates. A primary antibody against Nrf2 was then used to detect bound Nrf2. A secondary antibody conjugated to HRP provided a colorimetric readout at 450?nm with reference length at 655 nm (GENios, TECAN®). Results were normalized to protein concentration in each sample. Values are expressed as fold increase of each group compared to the sham group.

### Determination of caspase-9 activation

Caspase-9 enzyme activity was determined using a protocol adapted by Movsesyan et al. [36]. The assay is based on the hydrolysis of the p-nitroaniline (pNA) moiety by caspase-9. Brie?y, tissue lysates were incubated with assay buffer (50 mmol/L Hepes, pH 7.4; 0.2% CHAPS; 20% sucrose; 2 mmol/L EDTA; and 10 mmol/L dithiothreitol) and a 50 mmol/L concentration of chromogenic pNA speciﬁc substrate (Ac-Leu-Glu-His-Asp-pNA; Alexis Biochemicals, San Diego, CA, USA). In a ﬁnal volume of 100 μL (containing 60 μg of protein), each test sample was incubated for 3 h at 37 °C. The amount of chromogenic pNA released was measured with a microplate reader (GENios, TECAN®) at 405 nm. Values are expressed as fold increase of the mean of optical density (OD) of each experimental group compared to the sham group.

### RNA extraction

Total RNA from the hippocampus was isolated using Pure link RNA mini kit (Thermo Fisher Scientific, Life Technologies, Carlsbald, CA, USA), according to the manufacturer’s instructions. Briefly, hippocampal samples were homogenized in Lysis buffer with 1% β-mercaptoethanol by a homogenizer SHM1 (Stuart, Bibby Scientific LTD, Staffordshire, UK) on ice. Homogenized samples were added to an equal volume of 70% ethanol and mixed. The solution was passed through a filter cartridge, containing a clear silica-based membrane to which the RNA binds, and washed with Wash Buffer I and Wash Buffer II. RNA was finally eluted with RNase-free water and stored at -20 °C.

### RNA reverse transcription and real time PCR

Before reverse transcription, RNA was quantified using Nanoquant plate (TECAN). For each sample, 200 ng of total RNA were reverse transcribed using the High Capacity cDNA Reverse Transcription kit (Life Technologies), according to the manufacturer’s recommendations. Briefly, 10 µL of the sample were added to 10 µL master mix with RNase inhibitors and subjected to the appropriate thermocycling conditions. Finally, relative quantification by Taqman gene expression assay (Life Technologies) was performed by real time PCR (BIORAD CFX Connect) for the following genes: Nrf2 (Mm0047784_m1), GSTP1 (Mm04213618_gH), GSR (Mm00439154_m1), GSK3β (Mm00444911_m1) and as well as rn18S (Mm03928990_g1) and ACTB (Mm00607939_s1), as endogenous controls, using Universal Master Mix (Life Technologies). Each measurement was performed in triplicate and data were analyzed through the 2^-ΔΔCt^ method [37]. Sham mice were considered the calibrator of the experiment.

### Western blotting

Samples (30 μg proteins) were separated on 12% SDS polyacrylamide gels (Invitrogen, Carlsbad, CA, USA) and electroblotted onto 0.2 μm nitrocellulose membranes. Membranes were incubated overnight at 4°C with primary antibody recognizing Phospho-GSK3α/β (Ser21/9) (1:1000; Cell Signaling Technology Inc., Danvers, MA, USA) or HO-1 (1:1000; Enzo Life Sciences Inc., Lausanne, Switzerland). Membranes were washed with TBS-T (TBS +0.05% Tween20), and then incubated with a horseradish peroxidase (POD) linked anti-rabbit secondary antibody (1:2000; GE Healthcare, Piscataway, NJ, USA). Immunoreactive bands were visualized by enhanced chemiluminescence (ECL; Pierce, Rockford, IL, USA). The same membranes were stripped and reprobed with total GSK3α/β (1:1000; Cell Signaling Technology Inc.) or anti-β-actin (1:1000; Sigma-Aldrich, Saint Louis, Missouri, USA). Data were analyzed by densitometry, using Quantity One software (Bio-Rad). Values were normalized and expressed as fold increase of densitometry compared to the sham group.

### Immunohistochemistry

Fixed brains were sliced on a vibratome (Leika Microsystems, Milan, Italy) at 40 μm thickness and staining was assessed using the protocol described earlier [38].

#### Hematoxylin/eosin staining

Hematoxylin/eosin (H&E) staining was performed as described by Fischer et al. [39]. Briefly, selected section were mounted on slides and dried before dipping them in 100%, 95% and 70% ethanol (Sigma-Aldrich). Slices were washed and stained with hematoxylin for 8 min and then incubated for 10 min in tap water to promote the change to violet coloration. Subsequently, slices were washed in distilled water and then dipped 10 times in 80% ethanol before being immersed in 25% eosin solution (in ethanol 80%) for 1 min. Finally, slices were dehydrated in 95% and 100% ethanol solutions for 5 min before being fixed in xylen.

#### Caspase-9 staining

After deparafﬁnization, endogenous peroxidase was quenched with 3% hydrogen peroxide (H_2_O_2_). Non-speciﬁc adsorption was minimized by incubating the section in 10% normal goat serum for 30 min. Sections were then incubated overnight at 4°C with a rabbit anti-cleaved caspase-9 (1:500; Cell Signaling Technology Inc.), rinsed in TBS, and re-incubated for 1 h at room temperature with a goat biotinylated anti-rabbit IgG antibody (Vector Laboratories, Burlingame, CA, USA). Finally, sections were processed with the avidin-biotin technique and reaction products were developed using a commercial kit (Vector Laboratories). To verify the binding speciﬁcity, some sections were also incubated with only primary antibody (no secondary) or with the secondary antibody (no primary). In these situations, no positive staining was found in the sections, indicating that the immunoreactions were positive in all experiments carried out.

#### GFAP and Iba1 staining

After deparaffinization slices were washed in PBS and then incubated in TBS-A (TBS 0,1 % Triton-X 100) and then TBS-B (TBS-A 2% di BSA) to minimize non-specific absorption. Sections were then incubated overnight at 4°C with a mouse anti-GFAP primary antibody (1:300; Cell Signaling Technology Inc.) and a rabbit anti-Iba1 primary antibody (1:300; Wako Pure Chemical Industries, Osaka, Japan) in TBS-B with 3% Normal Goat Serum (NGS, Wako Pure Chemical Industries). Twenty-four hours later, slices were washed with TBS-A and TBS-B before the incubation with secondary anti-rabbit antibody (1:200; Alexa Fluor® 555, Life Technologies) and secondary anti-mouse antibody (1:200; Fluorescein, Life Technologies) in TBS-B with 3% NGS. To verify the binding speciﬁcity, some sections were also incubated with only primary antibody (no secondary) or with the secondary antibody (no primary). In these situations, no positive staining was found in the sections, indicating that the immunoreactions were positive in all experiments carried out.

#### Quantitative image analysis

Image analysis was performed by a blinded investigator, using an AxioImager M1 microscope (Carl Zeiss, Oberkochen, Germany) and a computerized image analysis system (AxioCam MRc5, Carl Zeiss) equipped with dedicated software (AxioVision Rel 4.8, Carl Zeiss). After deﬁning the boundary of the hippocampus at low magniﬁcation (2.5X objective), caspase-9, hematoxylin/eosin or GFAP and Iba1 staining were evaluated by densitometry of ﬁve different sections for each sample analyzed at a higher magniﬁcation (20X or 40X objective). Quantiﬁcation and morphological analysis were performed with the ImageJ software.

### Statistical analysis

Data were analyzed with the PRISM 5 software (GraphPad Software, La Jolla, CA, USA) and expressed as fold increase ± SEM of each group compared to the sham group. The difference between groups was analyzed one-way ANOVA with Bonferroni post hoc test. A difference was considered statistically signiﬁcant when a p value was less than 0.05.

## RESULTS

### CAPE ameliorated memory impairment after Aβ_1-42_O injection

**Figure 2:**
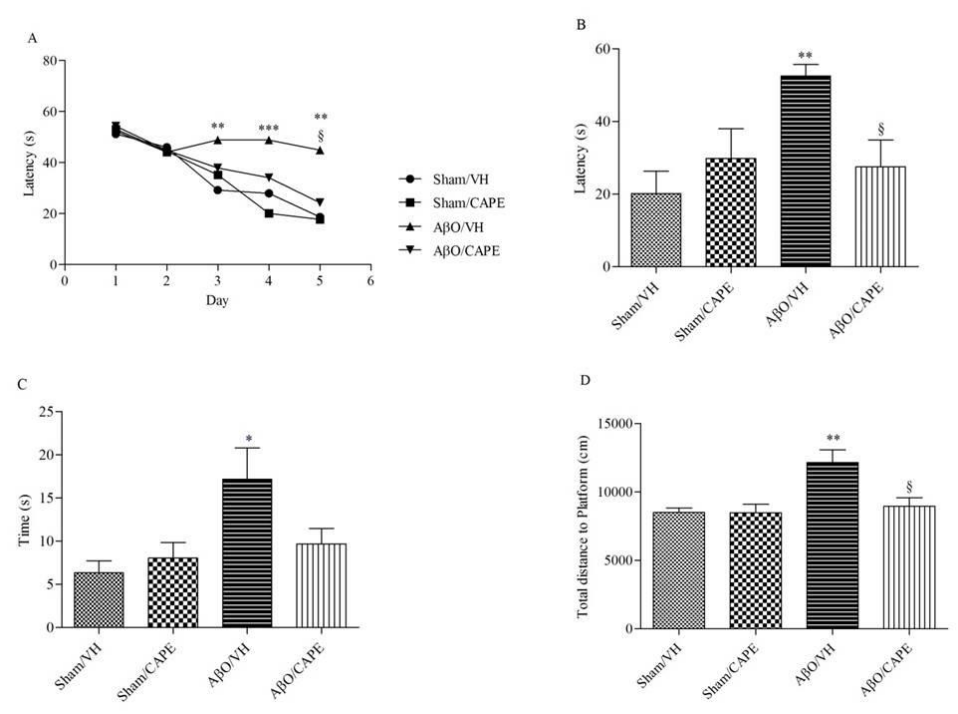
Effects of CAPE (10 mg/kg) on the performance in the training and probe trials of Morris Water Maze test in Aβ_1-42_O-injected mice The training trials (**A**) were carried out for 5 days (four per day), the probe trial was performed on day 6. Escape latency (**B**), time spent in the opposite quadrant to the platform zone (**C**) and total distance travelled from the platform zone (**D**) in the probe test. Values are expressed as mean ± SEM (n=10) (A: **p<0.01 vs. Sham/VH group, ***p<0.001 vs. Sham/VH group, §p<0.05 vs. AβO/VH; B: **p<0.01 vs. Sham/VH group, §p<0.05 vs. AβO/VH; C: *p<0.05 vs. Sham/VH group; D: **p<0.01 vs. Sham/VH group, §p<0.05 vs. AβO/VH; ANOVA, post hoc test Bonferroni).

We investigated whether CAPE could prevent Aβ-related cognitive impairments in mice. Our previous study showed that Aβ_1-42_O injection might mimic the pathological events in the early stage of AD patients [38]. MWM test was performed to investigate the effects of CAPE treatment on spatial learning and memory at days 11-16 after Aβ_1-42_O injection. At the last day of training, the escape latency was significantly different among various groups. The mice in the AβO/VH group took significantly more time to find the hidden platform as compared to the sham group, confirming that Aβ_1-42_O could induce impairments of spatial learning in mice (p<0.01, Fig. 2A). Moreover, the mice treated with CAPE spent a significantly shorter time finding the hidden platform as compared to the AβO/VH group, indicating that CAPE could attenuate Aβ_1-42_O-induced impairments of spatial learning (p < 0.05, Fig. 2A). In the probe trial, the platform was removed, and mice were allowed to swim freely. The mice in the AβO/VH group took more time to reach the platform location as compared to the sham group, suggesting that Aβ_1-42_O also caused impairments of spatial memory (p < 0.01, Fig. 2B). Interestingly, CAPE significantly reversed Aβ_1-42_O-induced impairments of spatial memory in mice, as demonstrated by the decrease of the escape latency, by the reduction of the time spent in the opposite quadrant to the platform zone and by the shorter total distance from the platform zone as compared to the AβO/VH group (p < 0.05, Fig. 2B-D). No alterations in swimming speed or total distance travelled were observed (data not shown).

**Figure 3:**
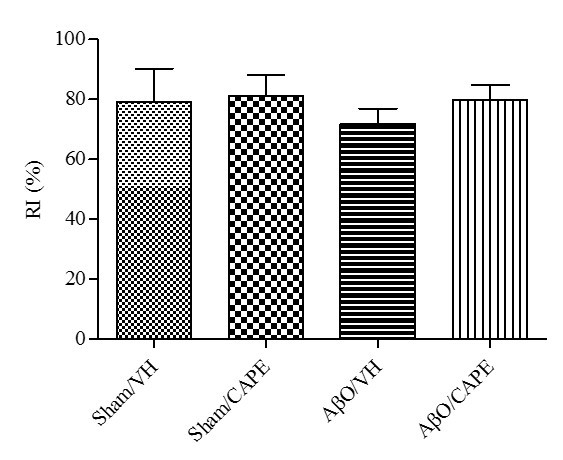
Effects of CAPE (10 mg/kg) on the performance in Novel Object Recognition test in Aβ_1-42_O-injected mice Quantitative comparison of the recognition index in the memory test session performed 24 h after the training session. Values are expressed as mean ± SEM (n=10).

We also evaluated recognition performance in mice at days 17-19 after injection of Aβ_1-42_O. A recognition index (RI), which is the percentage of time spent exploring the new object over the total time spent exploring the two objects, was determined as shown previously [38]. An RI of 50% is equivalent to the chance level, and a higher RI in the test phase indicates preferable object recognition memory. In the training session, the recognition index in various groups was not significantly altered (data not shown). The probe test did not show any significance among experimental groups; however, Figure 3 shows that AβO/VH animals spent less time in the exploration of the new object compared with Aβ/CAPE mice.

### CAPE counteracted Aβ_1-42_O-induced hippocampal cell loss

**Figure 4:**
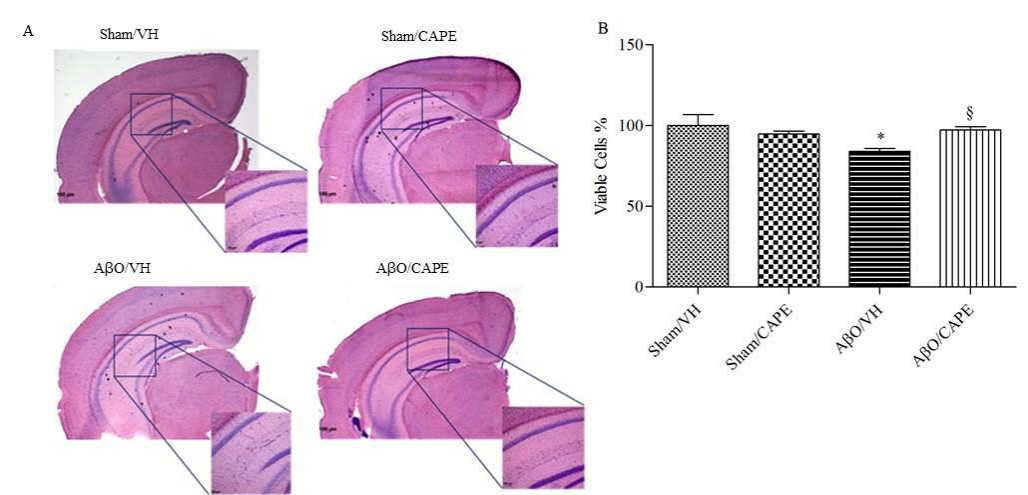
Effects of CAPE (10 mg/kg) on neuronal cell death 10 days after Aβ_1-42_O injection Representative hematoxylin and eosin staining of coronal sections containing the hippocampus. Scale bar 100 μm (A). Quantitative analysis of hematoxylin and eosin staining (B). Values are expressed as mean of fold increase ± SEM (n=10) of the density of each experimental group compared to the Sham/VH group (B: *p<0.05 vs. Sham/VH, ^§^p<0.05 vs. AβO/VH; ANOVA, post hoc test Bonferroni).

We evaluated the effect of CAPE on Aβ_1-42_O-induced hippocampal cell death using H&E staining. After behavioral analysis, mice were sacrificed and a significant decrease in the density of healthy neuron cells in CA1 region of hippocampus was observed in the AβO/VH group compared with the sham mice (p<0.05, Fig. 4). Typical neuropathological changes, including neuron loss and nucleus shrinkage or disappearance, were found in the CA1 of hippocampus in Aβ_1-42_O-injected mice, and were reversed significantly after CAPE treatment (p<0.05).

**Figure 5:**
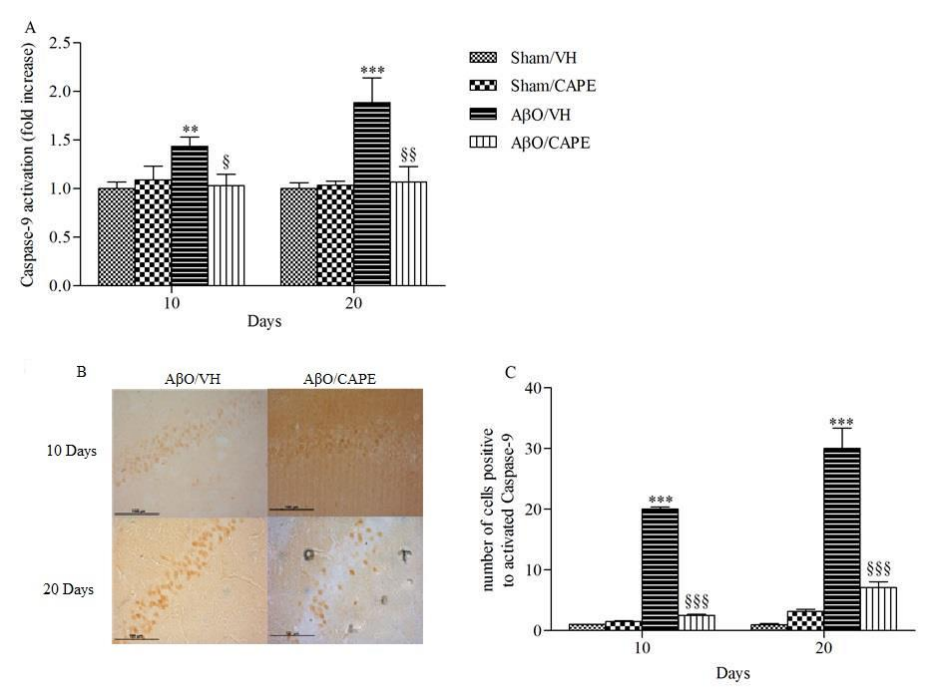
Effects of CAPE (10 mg/kg) on caspase-9 activation and immunoreactivity 10 and 20 days after Aβ_1-42_O injection A: Caspase-9 activation was determined using a speciﬁc chromogenic substrate in hippocampal samples. Values are expressed as mean of fold increase ± SEM (n=10) of optical density (OD) of each experimental group compared to the Sham/VH group. B: Representative photomicrographs of immunostaining for cleaved caspase-9 in brain coronal sections containing the hippocampus of AβO/VH and AβO/CAPE groups. Scale bar 100 μm. C: Quantitative analysis of the number of positive cells to caspase-9 activation. Values are expressed as mean ± SEM (n=10) of positive cells in each experimental group (A: **p<0.001 and ***p<0.001 vs. sham groups, ^§^p<0.05 and ^§§^p<0.01 vs. AβO/VH; C: ***p<0.001 vs. sham groups, ^§§§^p<0.001 vs. AβO/VH; ANOVA, post hoc test Bonferroni).

Caspase-9 is involved in the intrinsic signaling apoptotic pathway. This protease is known as a biomarker of oxidative stress-induced cell death, so we further investigated the effect of CAPE on its activation. As shown in Figure 5A, both at 10 and 20 days after Aβ_1-42_O injection, treatment with CAPE resulted in a significant (p<0.001) reduction in the activation of caspase-9, that returned to levels comparable to those of sham group. These findings were confirmed by immunohistochemical analysis. Figure 5B-C shows increased immunoexpression of activated caspase-9 in the AβO/VH group, when compared to the sham group. CAPE treatment was shown to be effective at inhibiting activation of caspase-9 induced by Aβ_1-42_O in the majority of hippocampal brain sections (p<0.001).

### CAPE attenuated the oxidative stress in the hippocampus of Aβ_1-42_O treated mice

**Figure 6:**
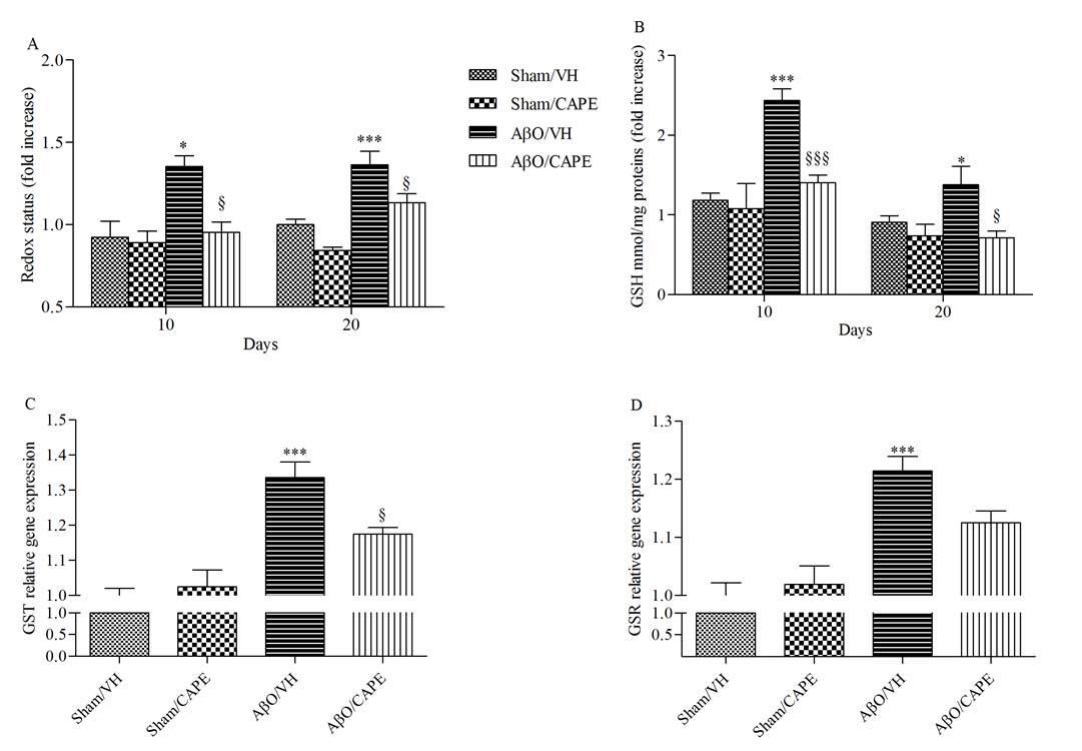
Effects of CAPE (10 mg/kg) on cellular redox status after Aβ_1-42_O injection Redox status was determined in hippocampal samples 10 and 20 days after Aβ_1-42_O injection (**A**) based on DCF’s fluorescence emission at 535 nm after excitation at 485 nm. Values are expressed as mean of fold increase ± SEM (n=10) of fluorescence intensity arbitrary units (UF) of each experimental group compared to the Sham/VH group. GSH content was measured using a colorimetric assay in hippocampal samples 10 and 20 days after Aβ_1-42_O injection (**B**). Values are calculated using a standard calibration curve and expressed as mean of fold increase ± SEM (n=10) of mmol GSH/mg protein compared to the Sham/VH group. GST and GSR mRNA relative expression (**C-D**) was determined in hippocampal samples 10 days after Aβ_1-42_O injection through the 2^-ΔΔCt^ method. Rn 18S and ACTB were used as control housekeeping genes, calculated through the 2^-ΔΔCt^ method and determined in hippocampal samples 10 days after Aβ_1-42_O injection. Values are presented as mean ± SEM of at least four different experiments (A: *p<0.05 and ***p<0.001 vs. sham groups, ^§^p<0.05 vs. AβO/VH groups; B: *p<0.05 and ***p<0.001 vs. sham groups, ^§^p<0.05 and ^§§§^p<0.001 vs. AβO/VH group; C: ***p<0.001 vs. sham groups, ^§^p<0.05 vs. AβO/VH group; d: ***p<0.001 vs. sham groups; ANOVA, post hoc test Bonferroni).

As shown in Figure 6A, Aβ_1-42_O injection caused obvious oxidant stress to the mice brain, indicated by significant increased ROS formation in the hippocampal samples (p<0.05 and p<0.001, 10 and 20 days after Aβ_1-42_O injection, respectively). Meanwhile, the levels of GSH in AβO/VH group were much higher than the sham group (Fig. 6B, p<0.001 and p<0.05, 10 and 20 days after Aβ_1-42_O injection, respectively). However, the administration of CAPE resulted in the significant decrease of ROS compared with the AβO/VH group. Moreover, with CAPE treatment GSH levels in the hippocampal samples of Aβ_1-42_O injected mice were decreased close to the sham group levels (Fig. 6B, p<0.001 and p<0.05, 10 and 20 days after Aβ_1-42_O injection, respectively). In addition, gene expression profiling has also been used as an effective biomarker to identify cellular stress. In the present study, gene expression analysis was performed for glutathione-S-transferase (GST) and glutathione reductase (GR) enzymes, showing that their mRNA expression levels were significantly (p < 0.001) higher in AβO/VH group, compared to the AβO/CAPE group (Fig. 6 C-D).

**Figure 7:**
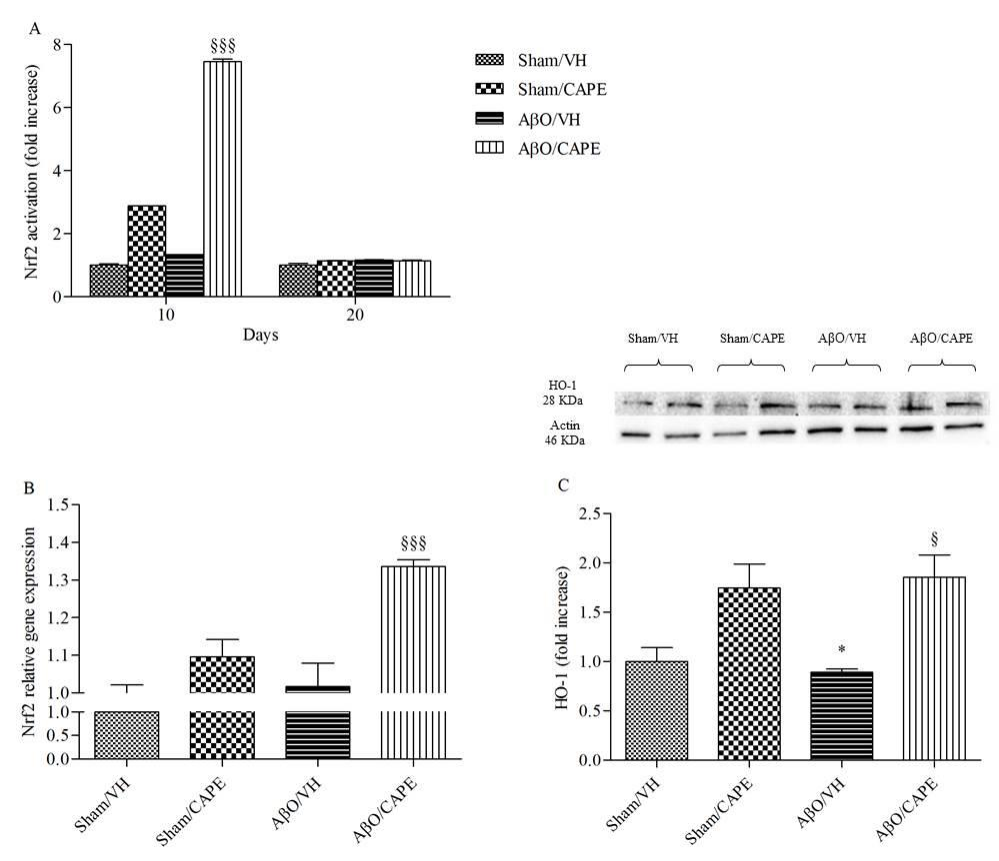
Effects of CAPE (10 mg/kg) on Nrf2 and HO-1 activation after Aβ_1-42_O injection Nrf2 activation was detected 10 and 20 days after Aβ_1-42_O injection using an Nrf2-based ELISA kit on nuclear extract of hippocampal samples (**A**). Values are expressed as mean of fold increase ± SEM (n=10) of the optical density (OD) of each group compared to the Sham/VH group. Nrf2 mRNA relative expression (**B**) was determined in hippocampal samples 10 days after Aβ_1-42_O injection through the 2^-ΔΔCt^ method. Rn 18S and ACTB were used as control housekeeping genes. Values are presented as mean ± SEM of at least four different experiments. HO-1 was determined 10 days after Aβ_1-42_O injection by Western Blotting at 28kDa and the loading control β-actin at 42kDa (**C**). Top: representative images of the protein expression. Bottom: quantitative analysis of the Western Blotting results for the HO-1 levels. The graphs show densitometry analysis of the bands appertaining to the protein of interest. Values are expressed as mean of fold increase ± SEM (n=10) of each group compared to the Sham/VH group (A: ^§§§^p<0.001 vs. AβO/VH group; B: ^§§§^p<0.001 vs. AβO/VH group; C: *p<0.05 vs. Sham/VH group, ^§^p<0.05 vs. AβO/VH group; ANOVA, post hoc test Bonferroni).

### CAPE modulated the Nrf2/HO-1 signaling pathway and GSK3β activity in the hippocampus of Aβ_1-42_O treated mice

Given the importance of the Nrf2 signaling pathway for regulating antioxidant responses in various models of neurodegenerative disorders [40], we examined the effects of CAPE treatment on Nrf2 signaling in the hippocampus of mice. ELISA analysis indicated that Nrf2 DNA-binding activities in nuclear fractions from the AβO/CAPE mice were increased compared with the AβO/VH group at 10 days post-Aβ_1-42_O injection (p<0.001, Fig. 7A). Moreover, qRT-PCR analysis indicated that the Nrf2 mRNA levels in AβO/CAPE mice were higher than those in AβO/VH mice at the same time point (p<0.001, Fig. 7B). To address the effectiveness of the CAPE-induced activation of the Nrf2 signal, we measured the protein expression of HO-1, an inducible and redox-regulated enzyme that counteracts the toxic effects of ROS [41]. The western blot analyses revealed a decreased expression of HO-1 (p<0.05) protein in Aβ_1-42_O-injected mice, whereas CAPE treatment significantly increased HO-1 protein levels (p<0.05) in the hippocampal samples at 10 days post-Aβ_1-42_O injection (Fig. 7C).

**Figure 8:**
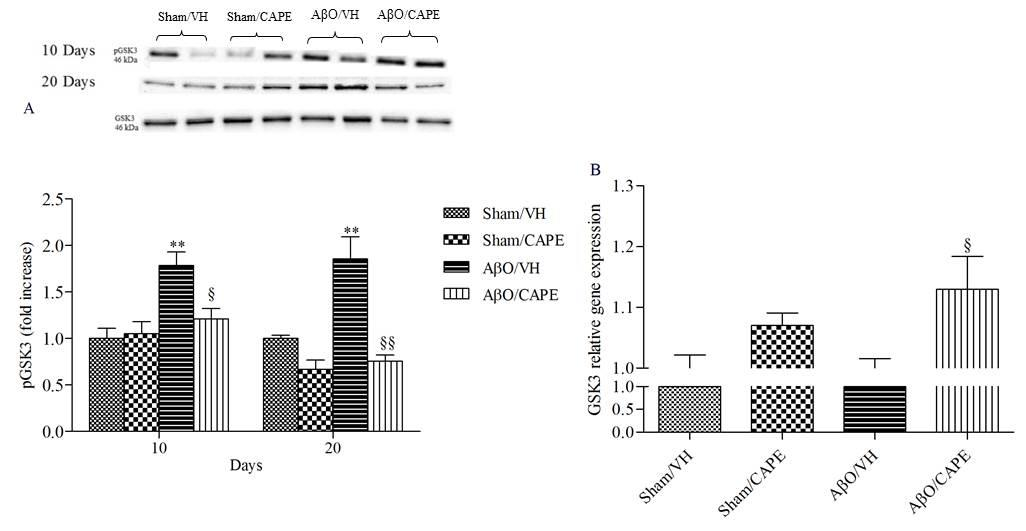
Effects of CAPE (10 mg/kg) on GSK3 phosphorylation (pGSK3) after Aβ_1-42_O injection pGSK3 was determined 10 and 20 days after Aβ_1-42_O injection by Western Blotting at 46kDa using total GSK3 as loading control (A). Top: representative images of the protein expression in hippocampus. Bottom: quantitative analysis of the Western Blotting results for the pGSK3 levels. The graphs show densitometry analysis of the bands appertaining to the protein of interest. Values are expressed as mean of fold increase ± SEM (n=10) of each group compared to the Sham/VH group. GSK3 mRNA relative expression (B) was determined in hippocampal samples 10 days after Aβ_1-42_O injection through the 2^-ΔΔCt^ method. Rn 18S and ACTB were used as control housekeeping genes. Values are presented as mean ± SEM of at least four different experiments (A: **p<0.01 vs. sham groups, ^§^p<0.05 and ^§§^p<0.01 vs. AβO/VH group; B: ^§^p<0.05 vs. AβO/VH group; ANOVA, post hoc test Bonferroni).

Increasing evidence suggests that the GSK3 activity is directly impacted by Aβ_1-42_O exposure and is altered in AD brains. To explore the effects of CAPE treatment on GSK3β activity, the amount of its phosphorylation was assessed by western blot studies. In agreement with the data from our previous study [38], Aβ_1-42_O induced a significant increase (p<0.01) of GSK3 phosphorylation on Ser9, which corresponds to its inactivity, as compared to sham mice (Fig. 8A). CAPE treatment reversed the effects of Aβ_1-42_O and significantly decreased (p<0.05 and p<0.001, 10 and 20 days post-injection) GSK3β inhibitory phosphorylation. This observation was also confirmed using qRT-PCR. GSK3β mRNA levels were increased in the hippocampal tissue from AβO/CAPE group when compared with the AβO/VH group at 10 days post-Aβ_1-42_O injection (Fig. 8B).

### CAPE reduced glial cell activation (microglia and astrocytes) in the hippocampus of Aβ_1-42_O treated mice

**Figure 9:**
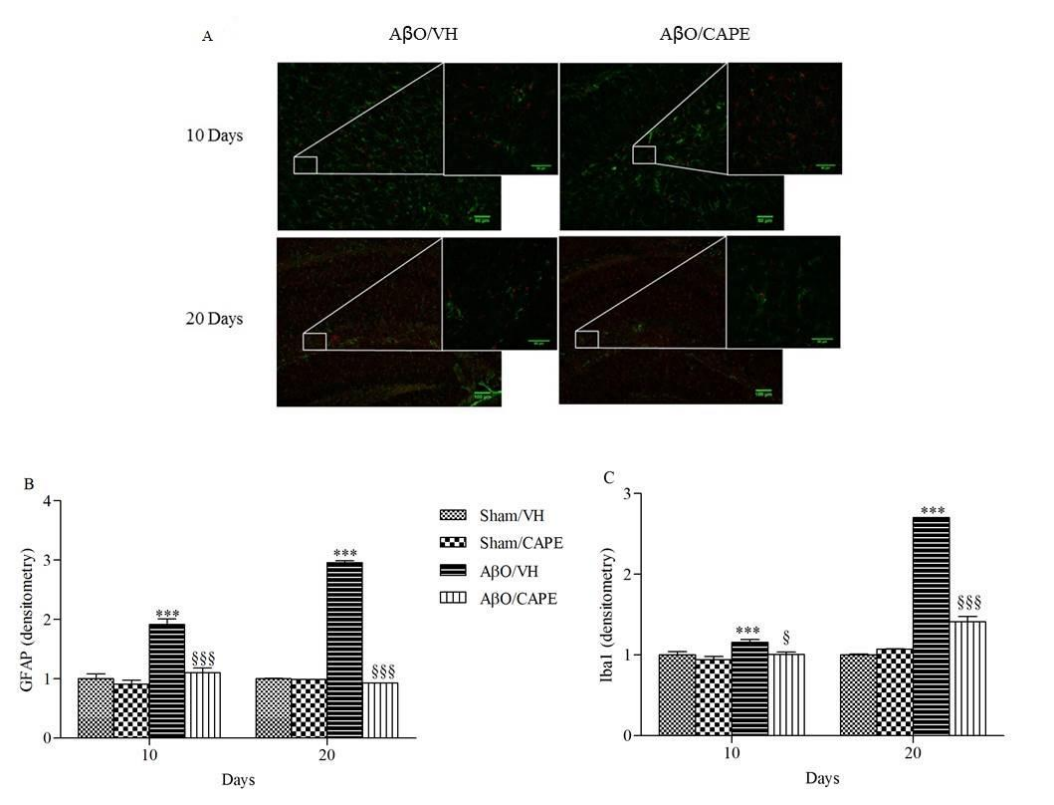
Effects of CAPE (10 mg/kg) on inflammatory response 10 and 20 days after Aβ_1-42_O injection Representative photomicrographs (**A**) of immunostaining for GFAP (green) and Iba1 (red) in brain coronal sections containing hippocampal structure of AβO/VH and AβO/CAPE groups. Scale bar 100 μm. Quantitative analysis of GFAP (**B**) and Iba1 (**C**) immunostaining. Values are expressed as mean of fold increase ± SEM (n = 10) of the fluorescent intensity of each experimental group compared to the Sham/VH group (B: ***p < 0.001 vs. sham groups; ^§§§^p<0.001 vs. AβO/VH group; C: ***p < 0.001 vs. sham groups; ^§^p<0.05 and ^§§§^p<0.001 vs. AβO/VH group; ANOVA, post hoc test Bonferroni).

Several studies have indicated that there is an increase in microgliosis and astrocytosis during aging that can contribute to neurodegenerative disorders such as AD [42, 43]. Glial fibrillary acidic protein (GFAP) and ionized calcium-binding adaptor molecule 1 (Iba-1) are specific markers for activated astrocytes and microglia, respectively. Therefore, we investigated the protective effect of CAPE against microglial (Iba-1 reactive cells) and astrocyte (GFAP reactive cells) activation. Immunofluorescence images in the hippocampus revealed a significant increase in the number of Iba-1 and GFAP (p<0.001, both 10 and 20 days) reactive cells in the AβO/VH group compared to sham mice. On the other hand, CAPE treatment significantly decreased the number of reactive Iba-1 (p<0.001) and GFAP (p<0.001) cells in the hippocampus of Aβ_1-42_O treated mice (Fig. 9), which returned to levels similar to those in the sham group.

## DISCUSSION

The present study is the first to provide evidence that CAPE administration (10 mg/kg, i.p., 10 days) attenuates Aβ_1-42_O-induced ROS, memory impairment, neuroinflammation and neurodegeneration in a mouse Aβ_1-42_O model. In this study, we found that CAPE significantly ameliorates cognitive deficits accompanied by reduced levels of ROS and neuronal death in brain tissues and increased Nrf2/HO-1 expression in Aβ_1-42_O-treated mice.

Aβ, the major component of senile plaques, is considered to have a crucial role in the development of AD [44]. The mechanisms by which Aβ causes neuronal toxicity remain elusive. However, extensive postmortem studies suggest that Aβ-induced oxidative stress is a pivotal cause of neuronal degeneration and death [45]. With age, genetic and environmental risk factors, the oxidative-redox system becomes imbalanced and oxidative stress ensues through increased levels of ROS. Unfortunately, the current drugs prescribed for AD show only a modest improvement in terms of symptomatic relief and delaying the progression of the disease. Thus, we hypothesize that increasing the intrinsic antioxidant defense might be a successful strategy to prevent oxidative stress-related neuronal pathologies, such as AD.

Clinical and experimental evidence has revealed that the acute increase of Aβ levels in the brain allows development of AD-like phenotypes [46, 47]. We have recently reported that a single i.c.v. injection of Aβ_1-42_O resulted in a significant impairment of memory function, imbalances in the redox status, apoptotic cell death and synaptic dysfunction. The i.c.v. administration of Aβ peptides into the mice brain is a valid model of early AD [38]. In the current study, our results showed a significant reduction in memory function as highlighted by MWM and partially by NOR test performance. We also observed that CAPE treatment improved memory, as shown by the reduction in escape latency, in the time spent in the opposite quadrant and by the shorter total distance from the platform zone during the probe test. These data are in agreement with the study by Kumar and coworkers [27] who demonstrated the ability of 28 days of CAPE treatment to halt the development of cognitive deficits in streptozotocin-i.c.v.-treated rats. In the NOR test, we did not observe any significance among experimental groups; however, AβO/VH animals spent less time in the exploration of the new object compared with AβO/CAPE mice. We hypothesized that the non-significance of this test can be attributed to the fact that the NOR task is based on the discrimination of familiarity of the stimuli, and for normal performance the subject needs to respond to “what” stimulus was used in the experiment previously [48]. Balderas and coworkers pointed out that the perirhinal, prefrontal and insular cortices consolidate the information of individual stimuli, i.e., objects, while the hippocampus consolidates the contextual information where the objects were experimented. In fact, hippocampus lesions impair spatial memory but not recognition memory and peri-postrhinal cortex lesions disrupt object recognition but not spatial memory. It is believed that soluble Aβ_1-42_O impairs long-term memory by degenerating the hippocampus. In fact, in our model mice showed hippocampus impairment as highlighted by the MWM test, and probably the NOR test probably failed to prove this deficit.

It is known that Aβ leads to massive and widespread neuronal cell death which is one of the major hallmarks of AD, along with abnormal mitochondria, NFTs and amyloid plaques [49]. Previous studies have shown that neuronal apoptosis is a key factor leading to neuronal loss, and neuronal loss in AD is closely linked with apoptosis [50]. The caspase family plays a very crucial role in mediating the process of apoptosis, the initiator caspase-9 then activates the executioner caspases, such as caspase-3, which brings about the apoptotic destruction of the cell. It has been proved that caspases not only mediate the process of apoptosis of cortical and hippocampal neurons of AD [51], but also relate with Aβ [52], Aβ precursor protein (APP) [53], and NFTs [54]. Therefore, finding how to inhibit the activity of caspases may be a successful strategy for the prevention and treatment of AD [55]. Interestingly, in our study Aβ_1-42_O injection significantly increased caspase-9 activity in the hippocampus, whereas it was dramatically lower in the mice treated with CAPE, suggesting that CAPE could abort the apoptotic signaling pathway. Our results are consistent with previous findings [56], which showed the ability of CAPE to completely repress caspase-9 activation in primary cultures of cerebellar granule neurons exposed to low K^+^ concentrations.

Oxidative stress is an important factor contributing to the initiation and progression of AD [57]. Increased levels of oxidative stress markers were found in neurons surrounding amyloid deposits in transgenic mouse models of AD [58], and the induction of oxidative stress leads to Aβ accumulation in primary neurons [59]. In the present study, CAPE alleviated Aβ_1-42_O-induced increase in levels of ROS in the hippocampus, confirming its already known antioxidant activity [60, 61]. Although the GSH content of neurons is low, it is the primary antioxidant defense of the brain and alterations of its levels have been observed in various neurodegenerative processes. As already shown [38], in our model GSH levels increased significantly in the hippocampus after Aβ_1-42_O injection in mice. This result is thought to explain a compensatory rise in the GSH-related antioxidant system in response to increased ROS formation, and it was also confirmed by the increase of GST and GR mRNA levels. Our findings are supported by Cetin and Dincer [62], who have shown that increased levels of GSH in temporal cortex and basal forebrain after intrahippocampal Aβ_1-42_ injection could be a protective mechanism due to oxidative stress. On the other hand, CAPE treatment maintained GSH levels in the hippocampus of Aβ_1-42_O injected mice close to the sham group, which can be attributed to its scavenger and antioxidant actions [63].

A growing body of literature suggested that the activation of Nrf2 provides neuroprotection in AD [64]. Considering that Nrf2 is a key redox-regulated gene and mediates the general antioxidant responses, it could be a potential therapeutic target for neurodegenerative diseases, where cells are in a chronic state of oxidative stress. As the downstream of the antioxidant defense system, the expression of HO-1 is closely associated with Nrf2. Indeed, the induction of phase II enzymes such as HO-1 has the potential to enhance cellular antioxidant capacity and ameliorate oxidative injury [65]. Consistently, we observed that CAPE increased the nuclear levels of Nrf2 after 10 days of i.p. treatment, which validated previous indications that Aβ-induced oxidative cell death was attenuated via activation of the Nrf2/HO-1 pathway [66]. Moreover, Aβ_1-42_O-treated mice exhibited decreased HO-1 expression, whereas CAPE treatment increased this expression in the hippocampus of Aβ_1-42_O-treated mice. It seems that the neuroprotective effect of CAPE against oxidative stress might rely on the Nrf2/HO-1 pathway.

Increasing evidence suggested that GSK3β activity is directly impacted by Aβ exposure and is altered in AD brains [67]. The “GSK3 hypothesis of AD” [68] proposed that the overactivation of GSK3β accounts for several features of this pathology such as memory impairment, tau phosphorylation, increased amyloid production, microglia-mediated inflammation, and neuronal death. However, direct evidence for this is still limited at present and some studies found no change in GSK3β activity [69] or reduced GSK3β activity [70] in AD. Paradoxical findings can probably attributed to the apoptosis-regulating actions of GSK3; it can, in fact, have opposite actions on apoptosis, either strongly inhibiting or promoting apoptotic signaling [71]. Interestingly, GSK3β phosphorylation (Ser9) was associated with up-regulation of antioxidant enzymes, in particular HO-1, and transient elevation of intracellular GSH in cells surviving acute stress, before occurrence of irreversible damage and death [72]. Our data showed that CAPE treatment reversed the effects of Aβ_1-42_O and significantly decreased GSK3β inhibitory phosphorylation; results also confirmed by mRNA GSK3β levels. We speculate that one possible function of this GSK3β activation induced by CAPE is to alert defenses against redox instability and coordinated redox changes, including HO-1 induction.

Neuroinflammatory processes, often associated with the induction of free radical generating enzymes and the accumulation of reactive astrocytes and microglial cells, are considered as a major source of oxidative stress. In prospective studies, anti-inflammatory treatments have delayed AD onset and alleviated or slowed cognitive decline [73]. AβO activate microglia, which causes inflammation in the brain and promotes the production of cytokines and other inflammatory mediators that might contribute to dysfunction, injury, and ultimately neuronal loss [74]. We showed that CAPE treatment significantly decreased the number of reactive microglia and astrocyte cells in the hippocampus of Aβ_1-42_O-injected mice. These data were confirmed by a recent study that proved that CAPE is a potent anti-inflammatory mediator that eliminates LPS-induced iNOS, COX-2, IL-1β and IL-6 expressions in microglial cells [75]. Given the pathogenic impact of oxidative stress and neuroinflammation, therapeutic strategies aimed at blunting these processes are considered an effective way to confer neuroprotection.

In summary, our results demonstrated that the reversal of cognitive deficits by CAPE treatment in Aβ_1-42_O-injected mice might be result of its antioxidant, antiapoptotic and antinflammatory activity. CAPE treatment was associated with increased expression of Nrf2 and HO-1, which are probably modulated by GSK3β activity. This unique mechanism explains, at least partially, the potent antioxidant capacity of CAPE, which might allow CAPE to succeed in treating AD where other ‘classic’ antioxidants have failed. Therefore, CAPE could be a potential candidate for further preclinical studies aimed at the treatment of cognitive impairment and dementia.
